# Pharmacokinetic and pharmacodynamic integration for optimal dosage of cefquinome against *Streptococcus equi* subsp. *equi* in foals

**DOI:** 10.1186/s13567-020-00853-2

**Published:** 2020-10-15

**Authors:** Dong-Ha Lee, Biruk Tesfaye Birhanu, Eon-Bee Lee, Seung-Jin Lee, Naila Boby, Yong-Soo Park, Seung-Chun Park

**Affiliations:** 1 Laboratory of Veterinary Pharmacokinetics and Pharmacodynamics, College of Veterinary Medicine, Kyungpook National University, Bukgu, Daegu, 41566 Republic of Korea; 2 Developmental and Reproductive Toxicology Research Group, Korean Institute of Toxicology, Daejeon, 34114 Republic of Korea; 3 Department of Equine Industry, Korea National College of Agriculture and Fisheries, Jeonju, 54874 Republic of Korea

**Keywords:** cefquinome, *Streptococcus equi* subsp. *equi*, antibacterial activity, PK/PD integration, optimal dosage

## Abstract

Cefquinome is administered in horses for the treatment of respiratory infection caused by *Streptococcus equi* subsp. *zooepidemicus*, and septicemia caused by *Escherichia coli*. However, there have been no attempts to use cefquinome against *Streptococcus equi* subsp. e*qui* (*S. equi*), the causative agent of strangles. Hence the objective of this study was to calculate an optimal dosage of cefquinome against *S. equi* based on pharmacokinetics and pharmacodynamics integration. Cefquinome (1.0 mg/kg) was administered by intravenous and intramuscular routes to six healthy thoroughbred foals. Serum cefquinome concentrations were determined by high-performance liquid chromatography. The in vitro and ex vivo antibacterial activity were determined from minimum inhibitory concentrations (MIC) and bacterial killing curves. The optimal dosage was calculated from the integration of pharmacokinetic parameters and area under the curve (AUC_24h_/MIC) values. Total body clearance and volume of distribution of cefquinome after intravenous administration were 0.06 L/h/kg and 0.09 L/kg, respectively. Following intramuscular administration, a maximum concentration of 0.73 μg/mL at 1.52 h (*T*_max_) and a systemic bioavailability of 37.45% were observed. The MIC of cefquinome against *S. equi* was 0.016 μg/mL. The ex vivo AUC_24h_/MIC values representing bacteriostatic, and bactericidal activity were 113.11, and 143.14 h, respectively. Whereas the %T > MIC for bactericidal activity was 153.34%. In conclusion, based on AUC_24h_/MIC values and pharmacokinetic parameters, cefquinome when administered by intramuscularly at a dosage of 0.53 mg/kg every 24 h, would be effective against infection caused by *S. equi* in foals. Further studies may be necessary to confirm its therapeutic efficacy in a clinical environment.

## Introduction

Cefquinome is a fourth-generation amino-thiazolyl cephalosporin used solely in veterinary medicine [1–3]. The chemical modifications of the basic cephalosporin structure provide cefquinome’s zwitterionic property that facilitates its rapid penetration across Gram-negative outer membranes, including the porins of the bacterial cell wall and broaden the antimicrobial activity spectrum compared with previous generation cephalosporins [2, 4, 5]. Cefquinome has been widely used for treating various infections in cattle and pigs [3]. In horses, cefquinome has been used for the treatment of respiratory tract diseases caused by *Streptococcus equi* subsp. *zooepidemicus*, and foal septicemia caused by *Escherichia coli*, with a recommended dose regimen of 1 mg/kg. However, there have been no prior reports using cefquinome as a treatment for strangles in horses.

Strangles (equine distemper) is a highly contagious upper respiratory tract disease of equines caused by *Streptococcus equi* subsp. *equi* (*S. equi*), which is a Gram-positive bacterium, belongs to β-hemolytic, Lancefield group C streptococci [6]. Strangles affects horses of all ages but is most common in weanling foals or yearlings, whose clinical symptoms are more severe. The penicillins have been considered the drug of choice for the treatment of strangles [7]. Besides, ceftiofur, ceftriaxone, cefotaxime and cefquinome have also shown to have high efficacy in vitro against strains of *S. equi* [8, 9]. Potentially, cefquinome would have therapeutic effects on infections caused by these strains. However, experimental evidence evaluating the effects of cefquinome against *S. equi* are limited, and there are no reported studies about using cefquinome in clinical circumstances for the treatment of strangles.

The pharmacokinetics (PK) of cefquinome have been studied in horses and various animals, including sheep, buffalo calves, camels, goats and laboratory animals [5, 10–15]. The optimal dose range of cefquinome has been suggested to be 1–10 mg/kg based on the minimum inhibitory concentration (MIC) and the PK parameters. However, designing the dosage regimens based on PK profiles only, and MIC as a sole parameter of the pharmacodynamic (PD) response, is insufficient because of the complex relationships between the concentration of antibiotics, bacterial susceptibility and the PD inhibitory effects against bacteria [16]. In order to bridge the gap, integration of PK parameters and multiple PD models, such as the maximum effect (*E*_max_) model, has been utilized to predict optimal dosages, which have been known to improve the clinical response to therapy and reduce the antimicrobial resistance [16]. Although studies regarding the integration of PK/PD of cefquinome have been conducted previously in some animals, there have been no previous attempts to suggest optimized dosage of cefquinome against *S. equi* in horses using PK/PD integration [17, 18].

Therefore, the objectives of this study were (1) to determine the concentrations of cefquinome in serum and PK parameters following intravenous (IV) and intramuscular (IM) administration at a dose of 1 mg/kg in horses using high-performance liquid chromatography (HPLC); (2) to provide the degree of serum protein binding; (3) to estimate the in vitro and ex vivo antibacterial activity of cefquinome against *S. equi*; and (4) to calculate the optimal dosage regimen for the infections caused by *S. equi* based on cefquinome PK/PD parameters.

## Material and methods

### Drugs and reagents

Cefquinome sulphate was purchased from Sigma (St. Louis, MO, USA). All reagents used for analysis in this experiment were HPLC or analytical grade and obtained from commercial sources: acetonitrile (ACN) and methanol (MeOH) (Duksan, Ansan, Korea), High Performance Liquid Chromatography (HPLC) grade water (Fisher ChemAlert Guide, Marietta, OH, USA) and trifluoroacetic acid (TFA; Sigma). Cefquinome sulphate (Cefa4 inj®) for IV and IM administration was purchased from Shinil Biogen Co., LTD. (Ayang, South Korea).

### Animals and experimental design

The experiments were performed with six healthy thoroughbred horses (age, 6 months to 1 year), weighed 186 ± 23.5 kg, from the Korea National College of Agriculture and Fisheries. The horses were acclimatized to the environment for three weeks before the start of the experiment. The animals were housed in shaded and ventilated individual stalls, and hay and water were provided ad libitum.

Prior to the experiment, a clinical exam including physical examination, heart rate, and rectal temperature was checked that the horses were healthy. In addition, animals did not receive any antibiotic treatment for one month prior to the experiment. A two-phase cross-over design was performed in six horses. In period one, three horses of the first group received 1 mg/kg (total volume of 2 mL per 50 kg) of cefquinome by IV administration through the jugular vein, and the other group received the same dose of cefquinome by IM administration in the neck region between the scapula, the cervical spine, and nuchal ligament. Two weeks of wash-out ensued to ensure complete excretion of the drug from the bodies. In the second phase, the route of administration reversed, and the jugular veins used for administration of the drug and sample collection were different.

All research protocols and animal experiments in this study were reviewed and approved by the Institutional Animal Care and Use Committee (IACUC) in Gyeongsangbuk-do, Republic of Korea (Gyeongbuk IACUC-81).

### Sample collection and preparation

Six milliliters of blood samples were collected from the left jugular vein into 10-mL plain tubes using direct stick method. A total of 10 blood samples per horse were collected before (0 h) and at 0.25, 0.5, 0.75, 1, 2, 4, 8, 12 and 24 h after the drug administration as described previously [19, 20]. The blood samples were kept at room temperature, and clot retraction was allowed. Serum samples were separated by centrifugation at 5,000 x *g* at 4℃ for 20 min, and then 3 mL of supernatant serum was separated and stored at -70℃ until analysis.

### Sample treatment for HPLC analytical method

Aliquots (200 μL) of the serum sample were pipetted into 1.5-mL eppendorf tubes. Subsequently, 400 μL of MeOH was added to the aliquots for deproteinization. The samples were agitated for 10 s using a vortex mixer and centrifuged at 5,000 x *g* for 10 min. Then, the supernatant of the extract was filtered through a 0.45-μm PTFE syringe filter (Advantec, Japan) and transferred into a fresh autosampler vial. Twenty microliters of supernatant were injected into the HPLC system.

### HPLC instrumentation and analysis conditions

An HPLC Agilent Technologies 1100 series (Santa Clara, CA, USA) Series system comprising a reverse-phase Eclipse Plus C_18_ column (particle size, 5 μm; 4.6 × 250 mm), a quaternary HPLC pump, an autosampler and a UV detector was used to measure the cefquinome concentration in serum samples. The column compartment temperature was kept at 40 °C. The mobile phase consisted of ACN and water containing 0.1% TFA.

The concentrations of cefquinome were determined using the method described by Uney et al*.* with minor modifications [21]. The method used a binary gradient condition with water containing 0.1% TFA as mobile phase A and ACN as mobile phase B. The time program of the gradient is presented in Table 1. The flow rate was 1.1 mL/min, and the injection volume was 20 μL. The detection was measured using a UV detector set at 268 nm. The Agilent Chemstation software program (Agilent Technologies) was utilized to analyze the data and control the HPLC system.

**Table 1:** Mobile phase conditions for high-performance liquid chromatography in vitro analysis of cefquinome

Time (min)	Flow rate (mL/min)	A (%)	B (%)
0	1.1	90	10
7	1.1	50	50
10	1.1	50	50
11	1.1	90	10
15	1.1	90	10

A is acetonitrile, and B is water containing 0.1% trifluoroacetic acid

### Validation of HPLC analytical method

**Figure 1:**
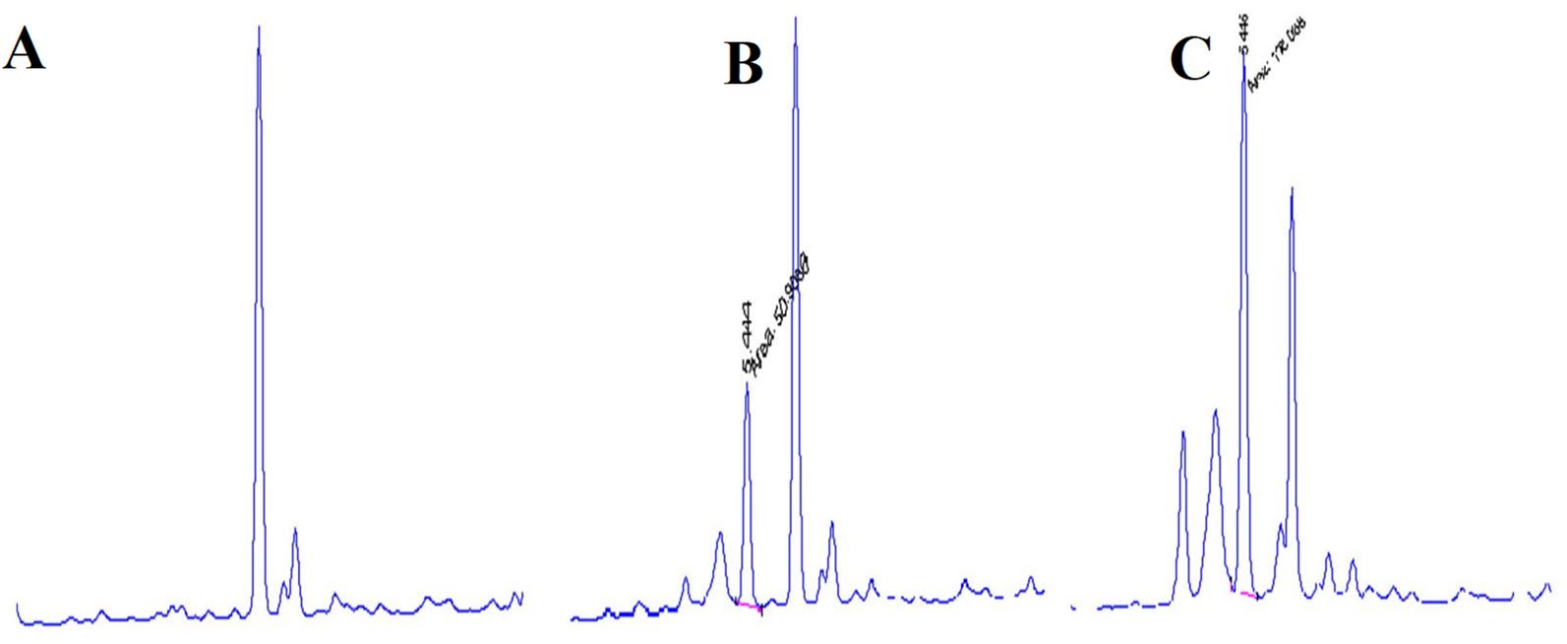
High-performance liquid chromatography chromatograms of cefquinome from horse serum (**A** blank, **B** spiked serum, **C** pooled serum sample)

A standard solution of cefquinome was prepared by the direct weighing of the standard substance with dissolution in sterile distilled HPLC grade water and appropriate buffer. The concentration of the standard stock solution was 1 mg/mL, and the stock solution was stored at −20 °C. The standard stock solution was diluted quantitatively to obtain concentrations of 0.8, 1.6, 3.2, 6.25, 12.5 and 25 μg/mL. After the stock solution had dissolved, blank serum samples from six horses that did not receive antibiotics were used to prepare the spiked samples at concentrations of 0.08, 0.16, 0.32, 0.625, 1.25 and 2.5 μg/mL, for calibration curves. Blank, spiked and pooled serum samples were analyzed to check for chromatographic interference peaks during the elution phase of cefquinome. Representative chromatograms of a blank serum, a spiked serum sample with 10 μg/mL of cefquinome, and a pooled serum sample collected at 15 min after IV administration are shown in Figure 1.

Linearity was determined in triplicate at different days by injections of six spiked serum samples ranging in concentration from 0.08 to 2.5 μg/mL. The calibration curve was represented by the following equation: *y* = *ax* ± *b*, where *y* is peak area; *x* is concentration (μg/mL). The detection and quantification limits were determined by the analysis of spiked serum samples. The limit of detection (LOD) was defined as the minimum level (lowest calibration standard) at which cefquinome was detected from background noise, but not quantifiable. The limit of quantification (LOQ) was defined as the lowest concentration of cefquinome analytes that could be measured with acceptable precision and accuracy [22].

Blank serum samples were spiked with cefquinome at a concentration range of 0.1–10 μg/mL and deproteinated with MeOH. After extraction of the analytes from the matrix and injection into the HPLC system, the recovery was determined by comparing the resulting peak areas with those of standard solutions with corresponding concentrations. Repeatability was studied for three injections of spiked serum samples at six different concentrations on the same day [22].

### Serum protein binding

The differences in the inhibition zone diameter between buffer solutions and blank serum samples were used to calculate the in vitro serum protein binding tendency of cefquinome, using the following equation described by Craig and Suh [23].

$${\text{Protein~binding~}}\left( {\text{\% }} \right) = \frac{{{\text{Zone~of~inhibition~(buffer)~{-}~Zone~of~inhibition~(serum)}}}}{{{\text{Zone~of~inhibition~(buffer)}}}} \times 100$$

The serum protein binding percentage of cefquinome at a concentration range of 0.16–20 μg/mL was estimated and presented in Additional file 1.

### Pharmacokinetics analysis

A computerized curve-stripping software program (WinNonlin, Certara, NJ, USA) was used to analyze the time versus serum concentration data after IV and IM administration of cefquinome for both one and two- compartmental models. The best-fitting model was chosen by the minimum Akaike’s information criterion estimation [24]. Normal (non-weighted) data were used for the analysis after comparing the distribution of the error around the lower and higher concentrations curve.

As a result, a biexponential equation was fitted to the serum concentration curves of cefquinome after a single IV administration. The PK parameters were calculated from the following equation: *C*_IV_(*t*) = *Ae*^(−*αt*)^ + *Be*^(−*βt*)^. *C*_IV_(*t*) is the concentration in serum at time (*t*) after IV administration; *Ae*^(−*α*t)^ represents the distribution phase and *Be*^(−*βt*)^ denotes the elimination phase. Based on this equation, the following variables were obtained: zero-time serum concentration intercepts of biphasic IV disposition curves (*A* and *B*); distribution and elimination rate constant (*α* and *β*); half-life of distribution and elimination (*α*_HL and *β*_HL); area under the curve (AUC); area under the first moment curve (AUMC); first-order transfer rate constants for drug distribution from the central compartment to the peripheral compartment and from the peripheral compartment to the central compartment (*K*_12_ and *K*_21_, respectively); mean residence time (MRT); volume of distribution at steady-state (Vd_ss_); total body clearance (CLB).

After a single IM administration of cefquinome, a mono-exponential equation was applied to the serum concentration curves of cefquinome. The PK parameters were determined from the following equation: *C*_IM_(*t*) = *A* × (e^−*K*10*t*^ − e^−*K*01*t*^). *C*_IM_(*t*) is the concentration in serum at the time (*t*) after IM administration; *A* is *D* × *K*_01_/*V* × (*K*_01_ − *K*_10_), where *D* is dose; *V* is the volume of distribution; *K*_01_ and *K*_10_ is first-order absorption and elimination rate constant, respectively. Based on this equation, the PK parameters, including *K*_01_ and *K*_10_, the half-life of absorption and elimination (*K*_01__HL and *K*_10__HL), maximum serum concentration (*C*_max_), the time required to achieve *C*_max_ (*T*_max_) and the AUC were calculated from the equation. Bioavailability (*F*) was calculated from the following equation: *F* (%) = AUC_IM_/AUC_IV_ × 100. All data obtained from the WinNonlin program was expressed as the mean ± standard deviation (SD).

### Determination of MIC

The strains *S. equi* (ATCC 39506) and *E. coli* (ATCC 25922), as a quality control microorganism, were used. Both microorganisms were purchased from the Korea Culture Centre of Microorganisms (KCCM). In order to determine the MIC for cefquinome, broth microdilution methods were performed, as outlined by the Clinical Laboratory and Standard Institute (CLSI) [25]. The bacteria cultures were grown freshly from beads stock stored at −70 °C, on tryptic soy blood (TSB) agar for *S. equi* and Luria–Bertani (LB) agar for *E. coli*. The standard inoculum was prepared by direct suspension in TSB and LB, respectively, and adjusted with sterile saline until the turbidity matched a 0.5 McFarland standard. The exact inoculum size was confirmed later via colony counts [22]. The bacterial cultures were diluted to approximately 2 × 10^6^ cfu/mL, and 100 μL of diluted bacterial suspension was inoculated into 96-well microplates, which contained serially-diluted cefquinome ranging from 0.001 to 256 μg/mL, and also, inoculated into drug-free control wells. The MIC was defined as the lowest concentration of drug at which no visible growth was determined visually by examination of the plates after 24 h of incubation at 37℃. In addition, the optical density (OD_600_) was determined using a VersaMax® microplate reader (Molecular Devices, Sunnyvale, CA, USA).

### Ex vivo bacterial killing curves

Standard inoculums were prepared, as described above, for MIC determination. The serum samples collected from horses prior to (0 h) and at 0.5, 1, 2, 4, 8, 12 and 24 h after IM administration of cefquinome were used to determine the ex vivo bacterial killing curves against *S. equi*. To 1 mL of each serum sample, 10 μL of stationary phase bacterial culture was added to give a final concentration of approximately 2 × 10^6^ cfu/mL and incubated. Aliquots (50 μL) were withdrawn from each culture tube at 1, 2, 4, 8, 12 and 24 h after incubation, and transferred for tenfold serial dilutions in 0.1% agar saline. Fifty microlitres of the suspension was then dropped onto quadrants of TSB agar. Once dried, the plates were incubated at 37 °C for 24 h to determine the viable counts (cfu/mL).

### PD analysis and PK/PD integration

The surrogate markers of antibacterial activity, that is, the AUC-to-MIC ratio (AUIC) and the duration during which the serum drug concentration exceeds the MIC (%*T* > MIC), were determined using in vitro PK parameters, MIC values and ex vivo PD parameters obtained from IV and IM administration of cefquinome. The inhibitory sigmoid *E*_max_ model, using the WinNonlin software program, was applied to calculate the ex vivo AUC/MIC ratio for the determination of the bacteriostatic (*E* = 0), bactericidal (*E* = −3) and bacterial elimination (*E* = −4) activities. As described previously by Aliabadi and Lees, the log_10_ difference between the bacterial count (cfu/mL) of the initial inoculum and the bacterial count at 24 h after incubation was fitted against the ex vivo AUC_24h_/MIC [26]. The values of ex vivo AUC_24h_/MIC were estimated by multiplying the measured serum concentration in samples collected between 0.5 and 24 h following IM administration of cefquinome and then dividing this value by the MIC [22]. The PD parameters were calculated using the following equation:

$$E = E_{0} - \frac{{E_{\max } \times C_{e}^{N} }}{{EC_{50}^{N} + C_{e}^{N} }}$$

where *E* is the antibacterial effect measured as the change in bacterial counts (log_10_ cfu/mL) in the serum sample at 24 h of incubation compared with the initial bacterial counts; *E*_max_ is the log_10_ cfu/mL difference in bacterial counts between 0 and 24 h in the drug-free serum sample; *E*_0_ is the log_10_ cfu/mL difference in bacterial counts in the test sample containing cefquinome at 24 h of incubation when the limit of detection (20 cfu/mL) is reached: *C*_e_ is the AUC_24h_/MIC ratio in the effect compartment (serum); *EC*_50_ is the AUC_24h_/MIC of drug require to produce 50% of the maximal antibacterial effect; *N* is the Hill coefficient, which describes the steepness of the AUC_24h_/MIC effect curve.

Based on the PK parameters and AUC_24h_/MIC values of the effect compartment, the optimal dosage was calculated using the following equation:

$${\text{Dose}} = \frac{{{\text{(AUC}}_{{{\text{24h}}}} {\text{/MIC)}} \times {\text{MIC}} \times {\text{Clearance}}_{{\text{(per hour)}}} { }}}{F \times fu}$$

where AUC_24h_/MIC denotes the values required to achieve bacteriostatic, bactericidal and bacterial elimination activity; MIC is the minimum inhibitory concentration; *F* is the bioavailability (see Sect. 2.8); *fu* is the free fraction of cefquinome.

The time during which the serum concentration exceeds the MIC (%*T* > MIC) was calculated as follows:

$$\% T > {\text{MIC}} = {\ln}\frac{{{\text{Dose}}}}{{{\text{Vd}} \times {\text{MIC}}}} \times \frac{{{\text{T}}_{{1/2}} }}{{{\text{ln2}}}} \times \frac{100}{\tau }$$

where ln is natural logarithm; Vd is the volume of distribution; *T*_1/2_ is the elimination half-life; *τ* is the dosing interval [27].

### Simulation of PK profile

Simulation of single daily doses of cefquinome for three consecutive days was calculated using previously reported half-lives of cefquinomes in horses. The dose selection achieved the AUC and Cmax over a 24 h dosing interval. The average ratio of the simulated AUC/MIC corresponds to Cmax that may exceed the MIC.

### Statistical analysis

The PK data was analyzed using Phoenix WinNonlin 8.1. The differences between IV and IM administration regarding the PK parameters (elimination half-life and AUC) were analyzed by a paired *t*-test, with *P* < 0.05 being considered significant.

## Results

### Validation of HPLC analytical methods

In the HPLC analytical method, the retention time of cefquinome was 5.45 min. No interfering peaks were detected in the blank sample during the elution phase of cefquinome. A linear relationship existed in the calibration curve between the cefquinome concentration and peak area (Additional file 2). The correlation coefficient (*r*^2^) was 0.9954 whereas, the equation was y = 19.448x + 0.9303. The validated LOD and LOQ for cefquinome were 0.05 and 0.16 μg/mL, respectively. The extraction recoveries were greater than 90%. The accuracy and precision values are summarized in Table 2.

**Table 2:** Accuracy and precision for Cefquinome in foal’s serum

Standard	Spiked (mean ± SD)	Precision	Accuracy
0.080	0.079 ± 0.002	2.5	99.987
0.156	0.16 ± 0.004	2.5	99.981
0.313	0.32 ± 0.012	3.9	99.965
0.625	0.61 ± 0.011	1.8	99.969
1.250	1.194 ± 0.012	1.0	99.954
2.500	2.39 ± 0.029	1.3	99.913

Values presented are mean ± SD of triplicate tests

### Serum protein binding

The protein binding rates of cefquinome in serum are shown in Additional file 1. The average in vitro serum protein binding rate over the concentration range of 0.15625–20 μg/mL was 3.91 ± 0.95%.

### Pharmacokinetics

**Figure 2:**
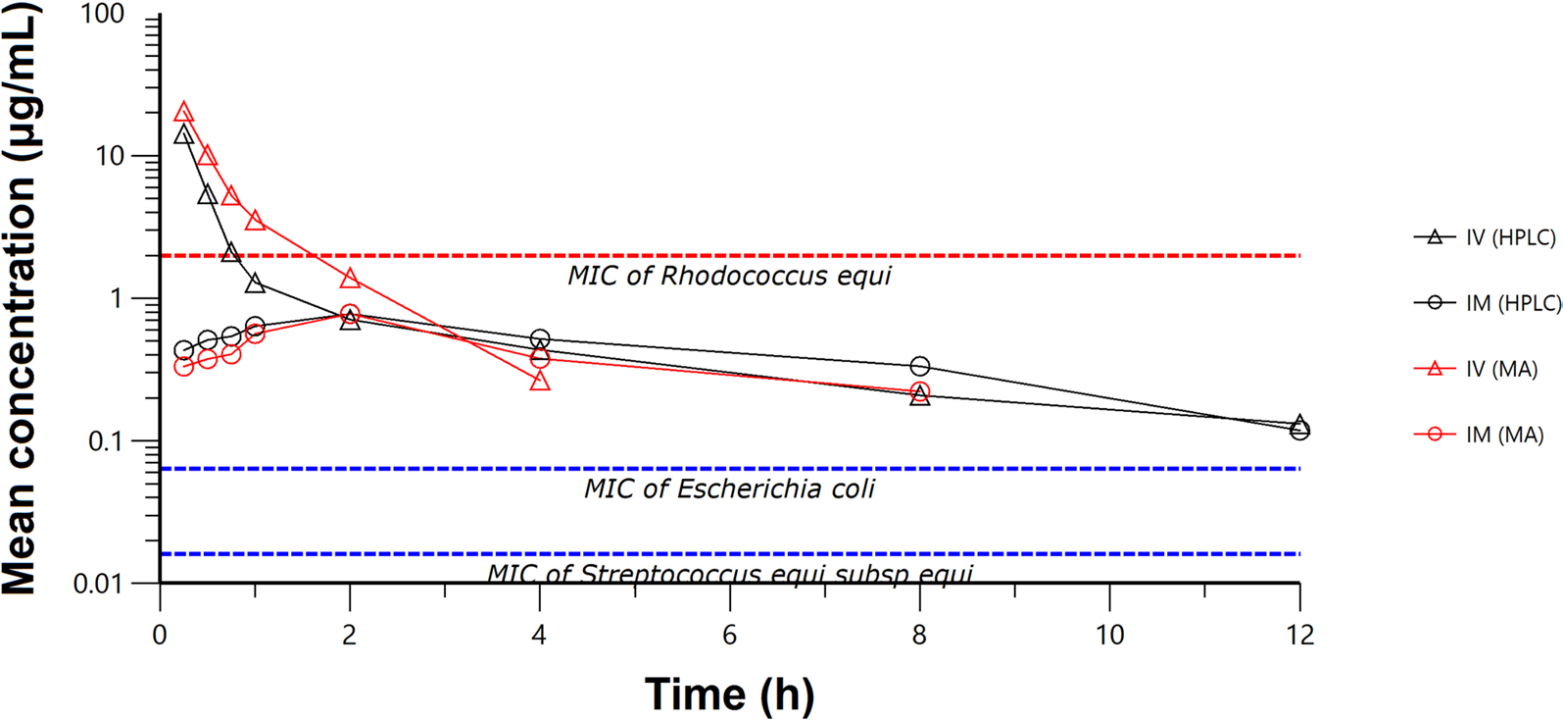
Semi-logarithmic graph of the time versus concentration in serum after a single IV and IM administrations of cefquinome in six horses analyzed by high-performance liquid chromatography and microbiological assay methods. The horizontal dashed line represents the MIC against *Streptococcus equi* subsp. e*qui* (0.016 μg/mL), *Escherichia coli* (0.032 μg/mL) and *Rhodococcus equi* (2 μg/mL)

The time versus cefquinome concentration relationships for six horses following IV and IM administration are shown in Figure 2. The data obtained after IV administration was described by a two-compartment model. Following IM administration, the data were found to fit a one-compartment model. The calculated PK parameters following IV and IM administrations of cefquinome to six horses are presented in Tables 3 and 4, respectively.

**Table 3:** Mean pharmacokinetic values (± SD) of cefquinome after IV administration of 1 mg/kg in horses (*n* = 6) determined by high-performance liquid chromatography

Parameter	Unit	Mean
*A*	μg/mL	55.89 ± 13.21
*α*	1/h	4.92 ± 1.59
*α*_HL	H	0.16 ± .08
*B*	μg/mL	1.25 ± .16
*β*	1/h	.25 ± .04
*β*_HL	H	2.77 ± .40
AUC	μg·h/mL	15.15 ± 1.47
AUMC	μg·h^2^/mL	21.82 ± 4.39
*K*_12_	1/h	1.44 ± .16
*K*_21_	1/h	0.37 ± .06
MRT	H	1.43 ± .21
Vd_ss_	L/kg	0.09 ± .01
CLB	L/h/kg	0.06 ± .01

*A* and *B* zero-time serum concentration intercepts of biphasic IV disposition curves, *α* distribution rate constant, *α*_HL half-life of distribution, *β* elimination rate constant, *β*_HL half-life of elimination, *AUC* area under the curve, *AUMC* area under the first moment curve, *K*_12_ and *K*_21_ first-order transfer rate constants for drug distribution from the central compartment to the peripheral compartment and from the peripheral compartment to the central compartment, respectively, *MRT* mean residence time, *Vd*_*ss*_ volume of distribution at steady-state, *CLB* total body clearance

**Table 4:** Mean pharmacokinetic values (± SD) of cefquinome after IM administration of 1 mg/kg in horses (*n* = 6) determined by high-performance liquid chromatography

Parameter	Unit	Mean
*K*_01_	1/h	1.73 ± .64
*K*_01__HL	h	0.45 ± .16
*K*_10_	1/h	0.16 ± .03
*K*_10__HL	h	4.39 ± .79
*C*_max_	μg/mL	0.73 ± .08
*T*_max_	h	1.52 ± .42
AUC	μg·h/mL	5.93 ± .55
*F*	%	37.45 ± 6.16

*K*_01_ first-order absorption rate constant, *K*_01__HL the half-life of absorption, *K*_10_ first-order elimination rate constant, *K*_*1*0__HL the half-life of elimination, *C*_max_ maximal serum concentration, *T*_max_ time taken to achieve maximal drug concentration, AUC area under the curve, *F* absolute bioavailability

After IV administration, the elimination half-life was 2.77 h (0.40). Cefquinome exhibited a low volume of distribution (Vd_ss_ of 0.09 L/kg), and the CLB was 0.06 L/h/kg.

Following IM administration, the elimination half-life was 4.39 h (0.79), which was longer than that after IV administration. The maximum serum concentration (*C*_max_) was 0.73 μg/mL, which was reached at (*T*_max_) 1.52 h. whereas the AUC obtained was 5.93 μg⋅h/mL. The mean bioavailability (*F*) after cefquinome administration was 37.45%.

### Pharmacodynamics

The MIC of cefquinome was determined in broth against *S. equi* (ATCC 39506) and *E. coli* (ATCC 25922) as the quality control strain, and the MIC values were 0.016 and 0.032 μg/mL, respectively. The serum concentration of cefquinome at 12 h was higher than the MIC of *S. equi* and *E. coli* (Figure 2).

**Figure 3:**
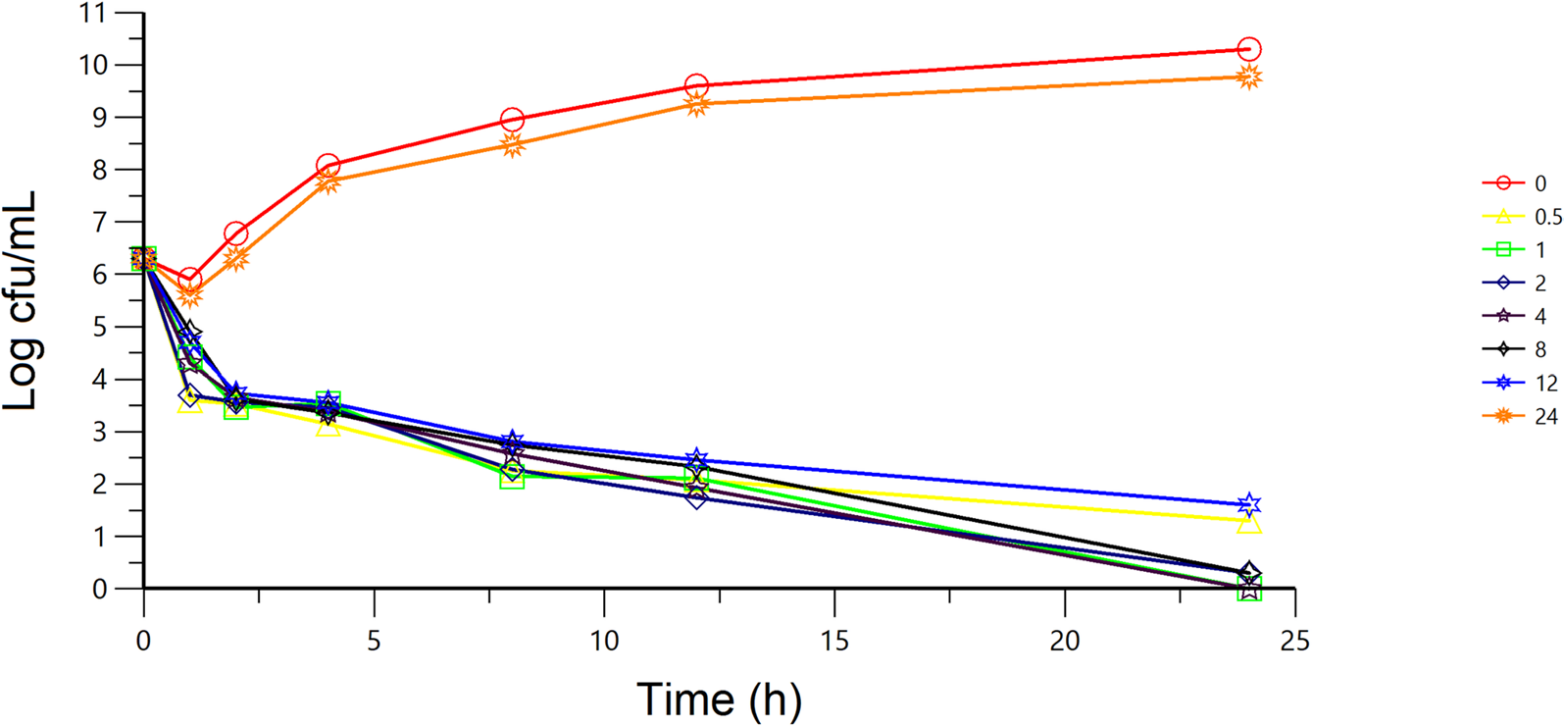
Ex vivo antibacterial activity of cefquinome against *Streptococcus equi* subsp. e*qui* after IM administration to six horses at a dose of 1 mg/kg

The ex vivo antibacterial activity of cefquinome, which showed a time-dependent antibacterial activity, was determined in serum against *S. equi* at predetermined time points using serum samples collected before the administration and at 0.5, 1, 2, 4, 8, 12 and 24 h after IM administration. A rapid bacterial inhibition activity was observed, and no bacteria were detected (detection limit 20 cfu/mL) after 24 h for serum samples collected between 1 and 8 h (Figure 3).

**Figure 4:**
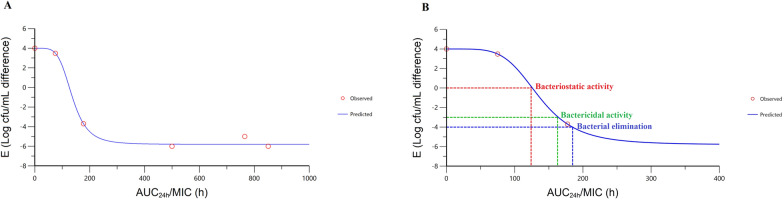
Sigmoidal *E*_max_ relationship for ex vivo AUC_24h_/MIC for *Streptococcus equi* subsp. e*qui* in horse serum samples versus bacterial count (log cfu/mL difference). The curve represents the line of predicted values from the inhibitory sigmoid *E*_max_ equation, and the circles are the mean observed values

The ex vivo AUC_24h_/MIC values for cefquinome in serum after IM administration of cefquinome at a dosage of 1 mg/kg are shown in Table 5. Furthermore, integration of the in vitro PK parameters, MIC and PD data from the inhibitory sigmoid *E*_max_ model provided the ex vivo AUC_24h_/MIC values required for various degrees of bacterial inhibition (Table 6). The relationship between the AUC_24h_/MIC values and bacterial counts is presented in Figure 4. The calculated AUC_24h_/MIC for serum that produces bacteriostatic (*E* = 0), bactericidal (*E* = -3) and bacterial elimination activity (*E* = -4) were 113.11, 143.14 and 159.16 h, respectively. In addition, the %*T* > MIC values based on the dose required for each bacterial inhibition effect were 147.17, 153.34 and 155.95% for 24-h dosing intervals.

**Table 5:** Ex vivo AUC_24h_/MIC values of *Streptococcus equi* subsp. e*qui* strain (mean ± SEM, *n* = 6) after IM administrations of cefquinome (1 mg/kg)

Serum samples (h)	AUC_24h_/MIC (h)	*E* (log cfu/mL)
0	0	4.39
.5	765 ± 112.41	−5
1	1277.5 ± 145.12	−6.3
2	1675 ± 182.23	−6
4	777.5 ± 170.35	−6.3
8	500 ± 78.04	−6
12	177.5 ± 16.43	−4.69
24	75 ± 17.54	3.48

**Table 6:** Pharmacokinetic–pharmacodynamic integration of ex vivo data after IM administration of cefquinome (1 mg/kg)

Parameter	Mean
*E*_max_ (log cfu/mL)	4.39
*E*_0_ (log cfu/mL)	−6.3
*E*_max_—*E*_0_ (log cfu/mL)	10.69
*EC*_50_ (log cfu/mL)	119.31
AUC_24h_/MIC for bacteriostatic activity (h)	113.11
AUC_24h_/MIC for bactericidal activity (h)	143.14
AUC_24h_/MIC for bacterial elimination (h)	159.16
Slope (N)	5.02

*E*_max_, difference in number of bacteria (cfu/mL) in blank serum sample between time 0 and 24 h; *E*_0_, difference in number of bacteria (cfu/mL) in pooled serum samples between time 0 and 24 h when the detection limit (20 cfu/mL) is reached; *EC*_50_, pharmacokinetic–pharmacodynamic parameter of drug that produced 50% of the maximal antibacterial effect; AUC_24h_/MIC, values required to achieve bacteriostatic, bactericidal and bacterial elimination activity; slope (N), the Hill coefficient that describes the steepness of the curve

### Optimal dosage calculation

The calculated dosages of cefquinome against *S. equi* for the bacteriostatic, bactericidal and bacterial elimination activity in this study were 0.38, 0.48 and 0.53 mg/kg, respectively.

## Discussion

The integration of the PK and PD indices are critical in estimating the efficacy and potency of a given drug, hence, are the basis for the selection of an applicable drug and to optimize dosage formulation [28]. The PK/PD integration of cefquinome has been described previously in various animals [12, 17, 18, 21, 29]. In the present study, we investigated to optimize the dosage against strangles caused by *Streptococcus equi* subsp. *equi*, which is a common disease in horses.

The degree of serum protein binding cefquinome varies between animal species, and various studies have shown that cefquinome exhibited a relatively low degree of serum protein binding, ranging from approximately less than 5–17% [30, 31]. Consistently, we have shown in this study that cefquinome represented a markedly low degree of protein binding (3.91%) in horse serum, which was similar to that in goats (11%) and sheep (8.254–13.002%) [11, 32, 33].

The disposition of cefquinome following IV administration in horses was best described by a two-compartment open model, with a rapid distribution and elimination phase (Figure 2). Experiments performed in other animals after IV administration have led to similar conclusions, except for a few PK parameters. After IV administration of cefquinome, the elimination half-life (*t*_1/2*β*_) of cefquinome in horses was 2.77 h, which was similar to the elimination half-life in piglets (1.85 h), sows (1.5–2.3 h), while a little higher than black swans (1.69 h) and dogs (0.85–0.98 h) [15, 29, 34, 35]. Moreover, the *t*_1/2*β*_ value was similar to that obtained from horses after IV administration of cephalexin (1.78 h), cefepime (2.1 h) and ceftriaxone (1.62 h) [36–38]. These relatively short half-lives suggest that cefquinome, as a cephalosporin drug, is rapidly eliminated in horses. Moreover, to avoid stress and acquainted with animal welfare, we set the initial time-point for taking blood sample after IV administration 15 min. According to the EPA guideline, the number of blood samples and the timing of sampling should be appropriate to allow adequate determination of absorption, distribution, and excretion [19, 20]. However, IV administration is considered to have a 100% absorption, which is not affected by the absorption phase. Furthermore, this experiment is designed to obtain AUC at each time, which is the most important PK parameter to calculate the optimal dosage.

The AUC of cefquinome following a single IV administration was 15.15 μg h/mL (1.47), which was higher than that reported in horses (6.16 μg h/mL), but lower than that obtained from female goats (43.57 μg h/mL) [14, 21]. The result was similar to the AUC obtained after IV administration of ceftiofur in foals (17.62 μg h/mL) but varies from horses (9.33 μg h/mL) [39]. Such differences are common and could be related to interspecies variation, assay methods, age, breed, dose and the drug formulation used.

The volume of distribution (Vd_ss_) indicates the diffusion of the drug in the body tissue. The Vd_ss_ of cefquinome in horses in this study was 0.09 L/kg. The Vd_ss_ value obtained from the current study was slightly lower than with those obtained from cephalexin (0.29 L/kg) and ceftriaxone (0.33 L/kg) in horses [36, 37]. Experimental investigations in other animals after IV administration of cefquinome have led to a similar conclusion, with values of 0.46 L/kg in piglets and 0.23 L/kg in calves, revealing that the Vd_ss_ of the cefquinome in the body is very small [29, 34]. The major reason for the low Vd_ss_ value appears to be the less hydrophobic nature of cefquinome, low fat-solubility and its low pKa value of 2.51–2.91, which effectively confines cefquinome to the extracellular fluid space [32].

In the present study, the disposition of cefquinome following 1 mg/kg of IM administration in horses was described by a one-compartment open model, which was similar to that described in buffalo, goat and camel [10, 12, 14]. However, the result disagreed with that reported previously in sheep, and chickens that administered cefquinome IM at a dose of 2 mg/kg in which the dispositions were described by two-compartment open models [5, 40].

After IM administration of cefquinome in horses, the elimination half-life of cefquinome was 4.39 h (0.79), which was similar to that in sheep (4.47 h), buffalos (3.73 h) and horses (3.15 h) following IM administration of 2.2 mg/kg ceftiofur [11, 12, 41]. Cefquinome showed long elimination half-life after IM injection in sheep, goats, buffalo calves, cattle calves, and cows. Horses that received cefquinome at 1 mg/kg also showed a relatively longer *t*_1/2*β*_ than those received 6 mg/kg [21]. Such differences are common and frequently related to interspecies variation, assay methods used, the formulation of the drug, and detection methods used [42].

In this study, the maximum serum concentration of cefquinome (*C*_*max*_) was 0.73 μg/mL. The result was similar to the *C*_max_ obtained from 1-month-old sheep (0.732 μg/mL) given IM administration of 1 mg/kg of cefquinome, and horses (0.885 μg/mL) following a single IM administration of 6.6 mg/kg ceftiofur crystalline-free acid [11, 43]. However, the result was lower than that obtained from horses after IV infusion of cefquinome (5.14 μg/mL) and IM administration of ceftiofur sodium in horses (4.49 μg/mL) [21, 44]. The time taken to achieve maximum serum concentration (*T*_max_) was 1.52 h, which was shorter than the *T*_max_ recorded in sheep (2.61 h) and goats (2.62 h) [42]. These variations might be attributed to anatomical differences between species, health status, and drug formulations in each case.

The systemic bioavailability (*F*) following IM administration of cefquinome was calculated to be 37.45%, which was noticeably smaller when compared with 89.31% in sheep, 86.30% in buffalo calves and 74.2% in black swans [5, 12, 35]. The low systemic bioavailability value of cefquinome in this study might be attributed to decreased absorption of the drug from the site of injection, low water solubility of the drug formulation, dispersion of the injected solution, and decreased blood flow at the muscle site [45, 46]. However, to overcome this problem, further studies on IM injections formulation is recommended.

The MIC of cefquinome against *S. equi* (ATCC 39,506) was 0.016 μg/mL in this study, which was consistent with the MIC against *S. equi* strains isolated from nine horses that ranged from 0.008 to 0.063 μg/mL [2].

The best PK/PD measures to describe the efficacy of time-dependent antibiotics is the time during which the drug concentration exceeds the MIC (%*T* > MIC). Cefquinome is a β-lactam antibiotic and so shows the time-killing characteristic, which means that %*T* > MIC is an essential parameter to describe the antibacterial activity. However, the AUC/MIC can also be considered as a better PK/PD index for β-lactams with long elimination half-life [47]. In general, though cefquinome has regarded as having short elimination half-life, it also shows long elimination half-life after intramuscular injection in sheep, goats, buffalo calves, cattle calves, and cows. Horses that received cefquinome at 1 mg/kg also showed a relatively long elimination half-life than those received 6 mg/kg [21]. Furthermore, Yu et al. argued that the different values of %*T* > MIC versus bacterial count (log cfu/mL) could not be obtained in ex vivo PK/PD integration, and the AUIC exerts a considerable influence on the treatment effectiveness of cefquinome [13, 18]. Such differences are common and frequently related to interspecies variation, assay methods used, and the formulation of the drug used [42].

In this study, the ex vivo AUC_24h_/MIC parameters were calculated, and the data was integrated by the inhibitory sigmoid *E*_max_ model with the differences between bacterial counts (log cfu/mL) after 24 h of incubation. The AUC_24h_/MIC required for bacteriostatic, bactericidal and bacterial elimination activity were determined in the serum and the optimal dose required to each effect compartment were 0.38, 0.48 and 0.53 mg/kg, respectively. However, these estimations did not consider factors, such as the host immune system and pathological changes during infection. In order to overcome these limitations, it would be necessary to consider estimates of PK parameters and MIC obtained from pathogenic species to accommodate the potential needs of the entire patient population [22].

**Figure 5:**
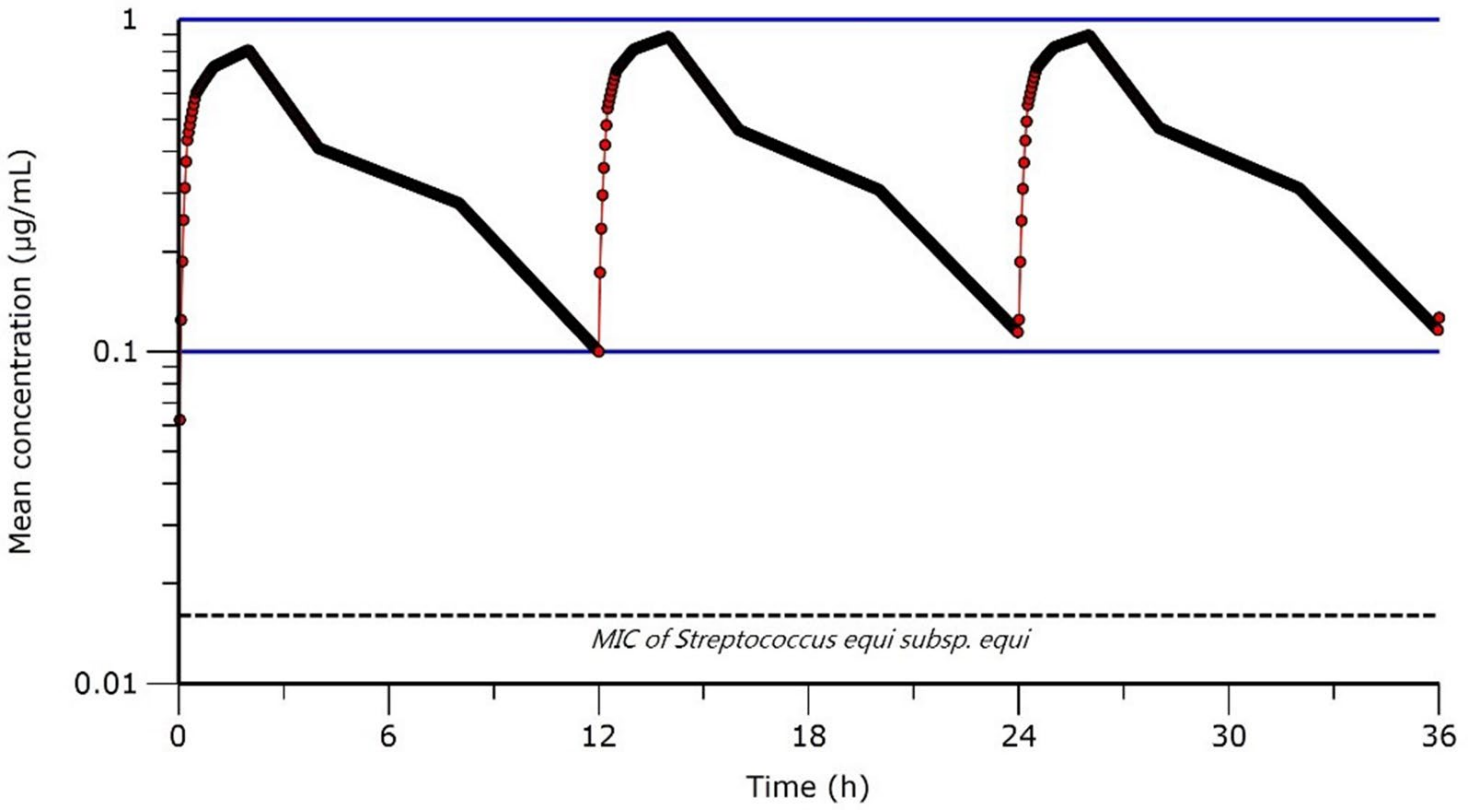
Simulated semi-logarithmic graph depicting the time versus cefquinome concentration in serum after repeated IM administration of the drug at a dose of 1 mg/kg every 12 h for three consecutive intervals. The indicated MIC of cefquinome against *S. equi* subsp. *equi* is 2 µg/mL

In order to evaluate the optimal daily dosage obtained from the current study, the equation from previous reports, in addition to the current data regarding the PK parameters of cefquinome, were used [48]. Considering the average serum cefquinome concentrations during the three consecutive dosing intervals (Figure 5), PK parameters were applied to calculate the daily IM dosage based on the following equation:

$${\text{IM dose of cefquinome}} = \left[ {\left( {C_{{({\text{average}})}} \times {\text{Vd}}_{{{\text{ss}}}} \times {24}\,{\text{h}}/{1}.{44}} \right)} \right]/T_{{{1}/{2}\beta }}$$

Accordingly, the calculated IM dosage was 0.175 mg/kg, which was three-fold lower than the dosage required to eliminate *S. equi* strain in this study. The reason it is different from the recommended dosage might be that the dosage regimens only based on PK parameters have limitations to reflect the characteristic differences of various strains, and the combination of PD parameters and PK/PD integration can be applied to overcome this limitation.

## Conclusions

This study is the first to use PK/PD modelling as a strategy for calculating the optimal dosage regimens of cefquinome against *S. equi*, the causative agent of strangles in equine species. The result suggests that the experimental dose administered either by IV or IM at a dosage of 1 mg/kg in horses is enough to exceed the MIC (MIC = 0.016 μg/mL) for up to 12 h in serum and that dosage of 0.53 mg/kg every 24 h could produce bacterial elimination activity. However, in this study we only used the Hill equation to determine the dosage using single values instead of using the Monte-Carlo simulations due to lack of sufficient MIC data range of cefquinome against *S. equi* subsp. equi in horses. Hence, there is a need to extend the current study by validating the calculated dosage in clinical circumstances to confirm its therapeutic efficacy.

## Ethics approval and consent for publication

All research protocols and animal experiments in this study were reviewed and approved by the Institutional Animal Care and Use Committee (IACUC) in Gyeongsangbuk-do, Republic of Korea (Gyeongbuk IACUC-81).

## Competing interests

The authors declare there is no conflict of interest.

## Supplementary information

**Additional file 1.** Serum protein binding percentage (%) of cefquinome in six horses.

**Additional file 2.** Calibration curves of cefquinome determined by high-performance liquid chromatography.

## Data Availability

The dataset(s) supporting the conclusions of this article is(are) included within the article.

## Abbreviations

AUC: area Under the Curve
CFU: colony Forming Unit
CLB: total body clearance
*C*_max_: maximum concentration
*E*_max_: maximum effect
F: bioavailability
HPLC: high-performance liquid chromatography
IM: intramuscular
IV: intravenous
KCCM: Korea Culture Centre of Microorganisms
LB: Luria–Bertani
MIC: minimum inhibitory concentration
MRT: mean residence time
PD: pharmacodynamics
PK: pharmacokinetics
TSB: tryptic soy blood
Vd_ss_: volume of distribution at steady state
