# Supplementary material for: Pharmacokinetic and pharmacodynamic integration for optimal dosage of cefquinome against Streptococcus equi subsp. equi in foals
Source: Vet Res. 2020 Oct 15;51:131. doi: 10.1186/s13567-020-00853-2 (PMC7566116; doi:10.1186/s13567-020-00853-2)
Supplement: Supplementary file 1 — Additional file 1. Serum protein binding percentage (%) of cefquinome in six horses. [file 13567_2020_853_MOESM1_ESM.docx]

**Additional file 1** Serum protein binding percentage (%) of cefquinome in six horses

| Concentration (μg/mL) | Protein binding percentage (%) |
| --- | --- |
| 0.15625 | 3.06 ± 2.21 |
| 0.3125 | 4.24 ± 1.94 |
| 0.625 | 5.00 ± 3.75 |
| 1.25 | 5.09 ± 2.68 |
| 2.5 | 4.99 ± 2.84 |
| 5 | 1.56 ± 1.71 |
| 10 | 3.80 ± .73 |
| 20 | 3.55 ± 3.28 |

Values presented are mean ± SD of triplicated test
