# Supplementary figures and images for: Pharmacokinetic and pharmacodynamic integration for optimal dosage of cefquinome against Streptococcus equi subsp. equi in foals
Source: Vet Res. 2020 Oct 15;51:131. doi: 10.1186/s13567-020-00853-2 (PMC7566116; doi:10.1186/s13567-020-00853-2)

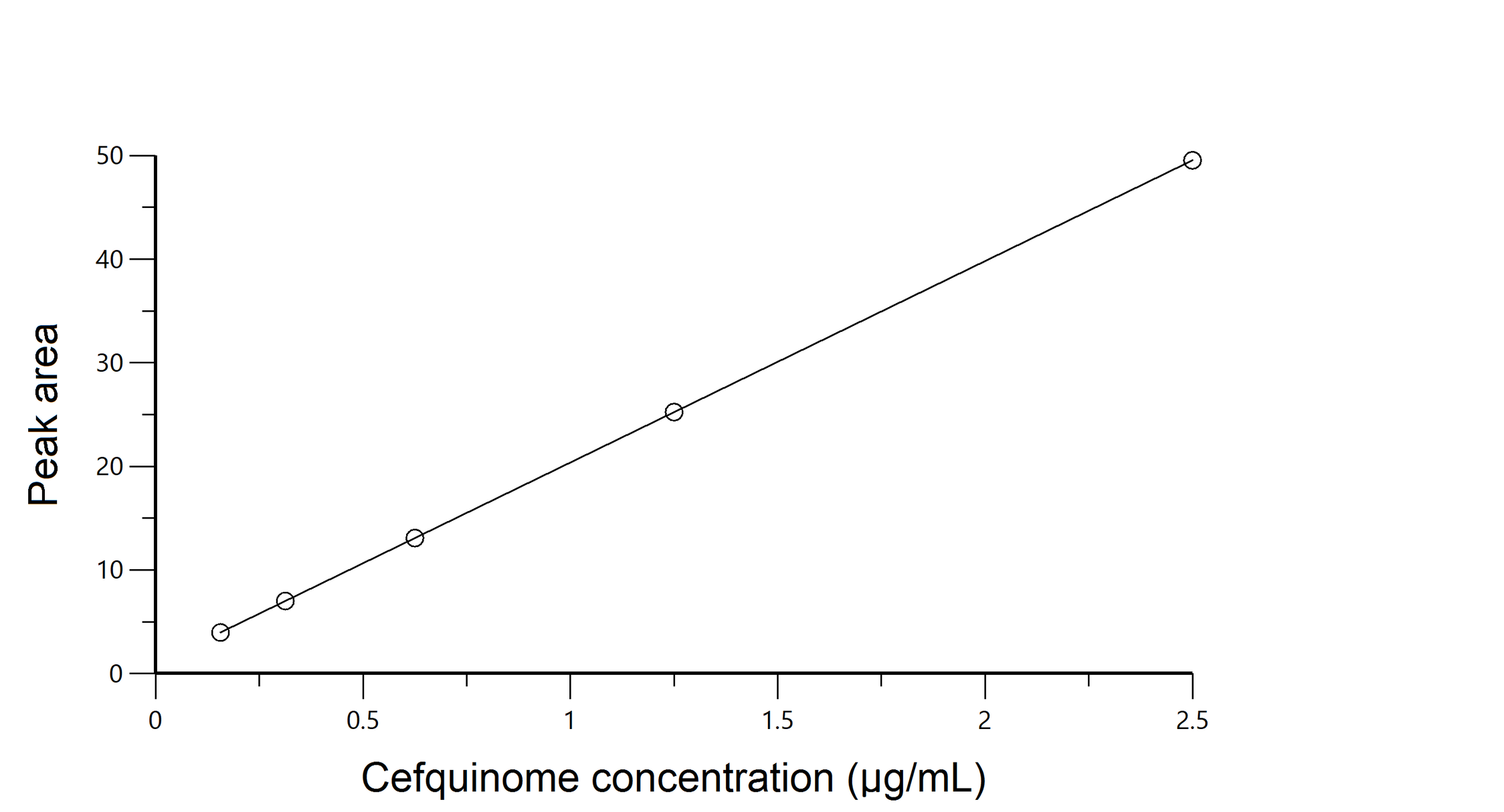

Supplement: Supplementary file 2 — Additional file 2. Calibration curves of cefquinome determined by high-performance liquid chromatography. [file 13567_2020_853_MOESM2_ESM.tif]
